# Eptifibatide bridging therapy for staged carotid artery stenting and cardiac surgery: Safety and feasibility

**DOI:** 10.1177/17085381221084813

**Published:** 2022-03-26

**Authors:** M Travis Caton, Kazim H Narsinh, Amanda Baker, Matthew R Amans, Steven W Hetts, Joseph H Rapp, James C Ianuzzi, Elaine Tseng, Warren J Gasper, Daniel L Cooke

**Affiliations:** 1 Neurointerventional Radiology, RinggoldID:8785 University of California San Francisco, San Francisco, CA, USA; 2 San Francisco Veterans Affairs Medical Center, San Francisco, CA, USA; 3 Vascular and Endovascular Surgery, RinggoldID:8785 University of California San Francisco, San Francisco, CA, USA; 4 Cardiothoracic Surgery, RinggoldID:8785 University of California San Francisco, San Francisco, CA, USA

**Keywords:** Eptifibatide, carotid stent, antiplatelet therapy, dual antiplatelet, carotid-coronary revascularization, endovascular surgery

## Abstract

**Background:**

Prophylactic carotid artery stenting (CAS) is an effective strategy to reduce perioperative stroke in patients with severe carotid stenosis who require cardiothoracic surgery (CTS). Staging both procedures (CAS-CTS) during a single hospitalization presents conflicting demands for antiplatelet therapy and the optimal pharmacologic strategy between procedures is not established. The purpose of this study is to present our initial experience with a “bridging” protocol for staged CAS-CTS.

**Methods:**

A retrospective review of staged CAS-CTS procedures at a single referral center was performed. All patients had multivessel coronary and/or valvular disease and severe carotid stenosis (>70%). Patients not previously on aspirin were also started on aspirin prior to surgery, followed by eptifibatide during CAS (intraprocedural bolus followed by post-procedural infusion which was continued until the morning of surgery). Pre- and perioperative (30 days) neurologic morbidity and mortality was the primary endpoint.

**Results:**

11 CAS procedures were performed in 10 patients using the protocol. The median duration of eptifibatide bridge therapy was 36 h (range 24–288 h). There was one minor bleeding complication (1/11, 9.1%) and no major bleeding complications during the bridging and post-operative period. There was one post-operative, non-neurologic death and zero perioperative ischemic strokes.

**Conclusions:**

For patients undergoing staged CAS-CTS, Eptifibatide bridging therapy is a viable temporary antiplatelet strategy with a favorable safety profile. This strategy enables a flexible range of time-intervals between procedures.

## Introduction

Perioperative stroke is a well-described complication of open cardiothoracic surgery (CTS), with an incidence ranging from 4.8 to 12.3% and resultant event-related mortality of 23%.^1–3^ Perioperative stroke etiology is multifactorial, but hemodynamically significant internal carotid artery (ICA) stenosis is an important pathophysiological mechanism of stroke in patients undergoing CTS.^3^ This presents a frequent clinical dilemma as up to 14% of patients referred for coronary artery revascularization also have radiographically severe ICA disease.^4,5^ Select patients with comorbid cardiac/carotid disease may therefore benefit from carotid revascularization *prior* to CTS.^1,6^

Over the past two decades, a spectrum of strategies has emerged to address cardiac and carotid disease contemporaneously.^7^ Among these, prophylactic CAS has proved an effective strategy to reduce perioperative stroke in patients with severe carotid stenosis who require CTS.^8^ Staging both procedures (CAS-CTS) during a single hospitalization (“staged” CAS-CTS) presents conflicting demands for antiplatelet therapy which must mitigate procedural complications of the CAS procedure but permit timely and safe sternotomy. At least five discreet antiplatelet strategies for staged CAS-CTS have been described in the literature.^9^ Each of these strategies is limited by the pharmacokinetics of oral antiplatelet agents (aspirin and thienopyridines) which can result in a “gap” in dual antiplatelet (DAPT) coverage during the period between stenting and sternotomy; the optimal pharmacologic strategy between procedures is therefore not clearly established.^9^

Eptifibatide (*Integrelin*), a parenteral glycoprotein (GP) IIb/IIIa antiplatelet agent with a half-life of 2.5 h, presents an alternate strategy to address the deficiency of oral DAPT strategies. Structurally a small peptide drug, eptifibatide mimics the tertiary structure of endogenous GPIIB/IIIA ligands such as fibrinogen, and thus disrupts the shared common pathway for platelet aggregation. Platelet function returns to normal within 4 h of discontinuation; thus, eptifibatide, contrasted with alternative to oral thienopyridines such as clopidogrel, enables rapid reversal prior to sternotomy.^10^ In addition, in the setting of coronary stenting, eptifibatide proved superior to immediate pre-procedural clopidogrel loading dose with respect to platelet inhibition.^11^ The versatility and efficacy of eptifibatide is recognized in a Class 1 American Heart Association (AHA) recommendation for use in patients with non-ST elevation myocardial infarction in whom percutaneous intervention is planned.^10^ Despite these favorable characteristics, eptifibatide has not yet been studied in the setting of staged CAS-CTS.

The purpose of this study is to present our initial experience with a “bridging” eptifibatide protocol for staged CAS-CTS to evaluate the safety and practical aspects of implementing this pharmacologic strategy in routine practice.

## Methods

A retrospective review of staged CAS-CTS procedures at a single referral center (San Francisco VA Medical Center). “Staged” CAS-CABG was defined by occurrence of the two procedures during a single hospitalization. Patients referred for coronary surgery and those referred for non-coronary cardiac surgery with cardiovascular risk factors were screened with carotid duplex ultrasound and/or CT angiography. Patients with symptomatic or asymptomatic severe ICA disease identified by carotid duplex imaging or CT angiography were evaluated for pre-CTS carotid revascularization with a threshold of ICA stenosis was >50% for symptomatic patients and >70% for asymptomatic patients using NASCET methodology. All patients were evaluated by a collaborative neurointerventional radiology and vascular surgery consultation and deemed appropriate candidates for CAS.

### Eptifibatide bridging protocol

All candidates for staged CAS-CTS not already on antiplatelet monotherapy were prescribed aspirin 81 mg daily for at least five days prior to CAS. All CAS were performed by a mixed-discipline team comprising an experienced vascular surgeon and neurointerventionalist. During the CAS procedure, therapeutic anticoagulation was achieved with an intravenous heparin bolus to achieve a target activated clotting time (ACT) of >250 s or double the baseline value. Eptifibatide was then administered as an intravenous bolus, at 90 μg/kg followed by a continuous infusion of 2 μg/kg/min for the duration of the CAS procedure. During the period between CAS and CTS (“interlude”), patients were admitted to the surgical intensive care unit and the eptifibatide infusion was continued along with daily aspirin until the morning of planned cardiac surgery. All CAS procedures were performed under monitored anesthesia care targeting minimal necessary sedation levels, using conventional transfemoral technique using distal embolic protection or flow-reversal and post-stent deployment balloon angioplasty. Patients were transitioned to oral DAPT with aspirin and clopidogrel the morning following CTS which was continued for 90 days followed by lifelong SAPT (daily aspirin 81 mg).

Post-operative (post-CTS) 30 -day neurologic morbidity and mortality was the primary endpoint. We also evaluated these outcomes during the “interlude” period, defined as the time between CAS and CTS. Endpoint indicators included Ischemic stroke which was defined by CT or MR confirmed lesion in the ipsilateral ICA territory with corresponding clinical deficit, and *transient ischemic attack* was defined as a radiographically negative, spontaneously resolving focal neurologic deficit.

## Results

### Patient cohort

Ten patients underwent 11 prophylactic CAS procedures as part of staged CAS-CTS during the study period. The mean age was 70 ± 7.1 years, and all patients were men. Nine of ten patients had radiographically severe (>70%) stenosis and one patient had symptomatic 60% stenosis. ICA stenosis was located at the origin/low cervical segment in 9/10 (90%) and the proximal petrous segment in one symptomatic patient. Four of ten (40%) of patients had at least moderate contralateral (>50%) stenosis and 2/10 (20%) had undergone previous endarterectomy of the contralateral ICA. Prevalent cardiovascular risk factors included current or prior tobacco use (5/10, 50%), hypertension requiring medication (8/10, 80%), hyperlipidemia (10/10/100%), and diabetes mellitus (2/10, 20%). Patient demographic details are outlined in Table 1.

**Table 1.** Patient characteristics.

Patient characteristics (*n* = 10)	
Age	70 ± 7.1 years
Sex	10/10 (100%) men
History of tobacco use	5/10 (50%)
Body Mass index	28.5 ± 5.4
Hypertension^a^	8/10 (80%)
Hyperlipidemia	10/10 (100%)
Diabetes mellitus	2/10 (20%)
*Indications for Cardiac surgery*	—
Multi-vessel CAD (CABG)	9/10 (90%)
Aortic stenosis (AVR)	3/10 (30%)
*Carotid disease classification*	—
*Symptomatic* (ipsilateral ischemic stroke or TIA within 6 months)	2/10 (20%)
*Asymptomatic*, remote ipsilateral ischemic stroke	1/10 (10%)
*Asymptomatic*, no prior ipsilateral ICA territory stroke	7/10 (70%)
NASCET severe stenosis (>70%)	9/10 (90%)
>50% contralateral ICA stenosis	4/10 (40%)
History of neck irradiation	1/10 (10%)
Prior contralateral CEA	2/10 (20%)

CEA = carotid endarterectomy, CAD = coronary artery disease, CABG = coronary artery bypass grafting, AVR = aortic valve replacement, ICA = internal carotid artery, TIA = transient ischemic attack, NASCET = North American Symptomatic Carotid Endarterectomy Trial.

^a^with current antihypertensive use.

### Carotid artery stenting

The technical success rate of 11 CAS procedures was 100% and there were no intraprocedural complications. All cervical interventions were performed with embolic protection technique. There were no adverse reactions or immediate complications on administration of eptifibatide during the CAS procedure. Technical details of CAS are summarized in Table 2.

**Table 2.** Procedural technique CAS and CTS.

Procedural technique CAS and CTS	
Carotid stent type	—
XACT (Abbott)	7/11 63.6%
Wallstent (Boston Scientific)	1/11 (9.1%)
Acculink (Abbott)	1/11 (9.1%)
Herculink (Abbott)	1/11 (9.1%)
Embolic protection device or flow reversal	10/11 (90.1%)
CAS technical success	11/11 (100%)
CAS intraprocedural complication (major or minor)	0/11 (0%)
Median delay, CAS to CTS	36 (range 24–288) hours
CABG bypass technique	—
“On pump”	8/9 (88.9%)
“Off pump”	1/9 (11.1%)
CABG intraprocedural complications	1/9 (11.1%)^b^
AVR intraprocedural complications	0/3 (0%)

^b^Intraoperative MI, requiring second round of cardiopulmonary bypass.

#### Bridging/interlude period

The duration between CAS and CTS (“interlude”) varied from 24 to 288 h with a median delay of 36 h. Variation of the interlude period facilitated logistical coordination between endovascular and cardiothoracic teams. There were no ischemic strokes during the interlude period. One minor bleeding complication occurred (1/11, 9.0%, groin hematoma, managed conservatively without transfusion) and zero major bleeding complications occurred (Table 3).

**Table 3.** Interlude and post-operative clinical outcomes.

Interlude and post-operative clinical outcomes	
Median follow-up	3.9 years (range 21 days–10.4 years)
Interlude (between CAS and CTS) complications	
Myocardial infarction	0/10 (0%)
Ischemic stroke	0/10 (0%)
Mortality	0/10 (0%)
Major bleeding complications	0/10 (0%)
Minor bleeding complications^+^	1/10 (10%)
Perioperative (30-day post-CTS) outcomes	
Myocardial infarction	1/10 (10%)
Ischemic stroke or TIA	0/10 (0%)
Mortality	1/10 (10%)
Surgical complications	2/10 (20%)
Surgical site infection	1/10 (10%)
Post-operative arrest, re-closure	1/10 (10%)
Perioperative (30-day post CTS) hemorrhagic complications	
Major bleeding complications	0/10 (0%)
Minor bleeding complications^c^	0/10 (0%)

^c^groin hematoma, no additional intervention.

#### Cardiothoracic surgery

**Figure 1.**
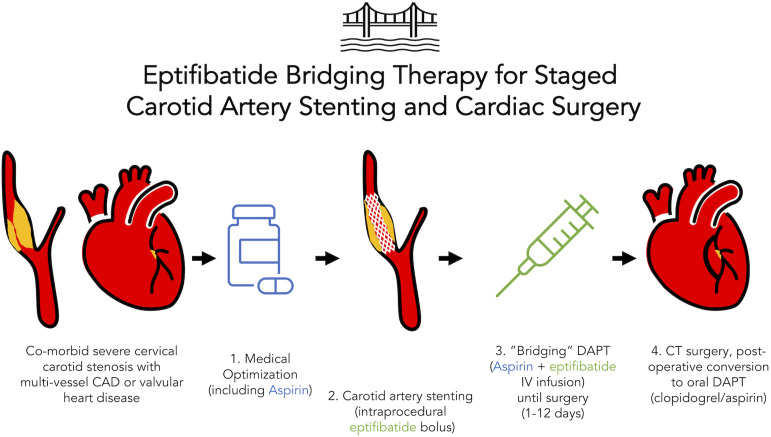
Schematic of the eptifibatide bridging protocol for staged CAS and CTS. CAD = coronary artery disease, DAPT = dual antiplatelet therapy, CT = cardiothoracic).

The indication for CTS was symptomatic multi-vessel coronary artery disease (CAD) in 9/10 (90%) and aortic stenosis in 3/10 (30%; two patients underwent combined coronary artery bypass (CABG) and one patient underwent isolated aortic valve replacement (AVR). Of the patients who underwent CABG, 8/9 (88.9%) were on-pump and one patient was off-pump. There was one intraoperative complication during CABG (intraprocedural myocardial infarction, supported on extracorporeal membrane oxygenation) and zero intraoperative complications during AVR (Figure 1). There were two post-operative surgical complications (one sternotomy infection requiring washout, one cardiac arrest on post-operative day one, which prompted re-do sternotomy and exploration but no additional coronary revascularization).

#### Outcomes

In the 30 -day post-CTS period there were no primary endpoint events (ischemic stroke). One of 10 patients died in the post-operative period (1/10, 10%) due to multiorgan failure on extracorporeal membrane oxygenation. There were no post-operative MI and no bleeding complications (major or minor) after CTS.

The overall mean follow-up duration was 4.75 years (range 21 days to 10.4 years). Of the nine patients alive beyond the 30 -day post-operative period, five were alive at last follow-up without ischemic stroke over a mean follow-up of 2.75 years and four patients died (1/4 heart failure, 1/4 cancer, 2/4 of undocumented cause) over a mean period of 5.71 years.

## Discussion

This study describes the feasibility, safety, and short-term results of a bridging eptifibatide antiplatelet protocol for patients undergoing staged CAS-CTS. In this small series, the bridging protocol was associated with no inter-procedural or post-surgical ischemic strokes and a low rate of minor bleeding complications.

The principal advantage of an antiplatelet bridging protocol in staged CAS-CTS is that it enables variable timing of the second stage CTS operation, an option which offers greater flexibility than conventional antiplatelet strategies for patients undergoing staged CAS-CTS. This flexibility is a particularly attractive feature for patients who live in remote or rural areas when a single, comprehensive hospital admission for both treatments is preferred. Staged CAS-CTS also affords more logistic degrees of freedom of scheduling among surgical and endovascular teams, whereas so-called “hybrid” procedures (CAS or CEA followed by CTS, same day) demands simultaneous availability of both teams. In addition, a flexible interlude period allows for medical optimization and recovery between the procedures as the between-procedure mortality for medically complex patients may be as high as 2.2%.^3^ Dong et al., in a recent study of 323 consecutive CAS-CTS patients, reported that “interlude” duration (time between procedures) of <5 days was associated with the primary composite endpoint, which suggests that the option of longer interlude duration may be appropriate for medically high-risk patients.^12^

The choice of eptifibatide as a second antiplatelet offers several key advantages over conventional DAPT protocols based on an aspirin/clopidogrel combination. Eptifibatide, along with GIIBIIIA agents abciximab and tirofiban, emerged in the early 2000s in several large, randomized trials for coronary intervention, though there is relative paucity of data for its use in CAS.^13^ When employed as an adjuvant to conventional DAPT, a small randomized trial found no incremental benefit of GIIBIIIA in CAS.^14^ Its use during routine CAS subsequently *declined* over time as there was paucity of clear benefit, particularly as the use of distal embolic protection became widespread.^15,16^ More recently, eptifibatide has been employed as a “rescue” therapy in acute CAS or intracranial procedures, owing to its rapid onset and profound inhibitive effect on platelet aggregation.^17^ Perhaps the most crucial advantage afforded to surgeons by eptifibatide is the ability to operate almost immediately after drug cessation; in the PURSUIT trial, CTS was proven safe within as little as two hours of eptifibatide cessation.^18^ These authors also found higher post-operative platelet counts, suggesting “platelet-sparing” effect during cardiopulmonary bypass. In addition, eptifibatide may overcome the challenge of pharmacologic resistance to clopidogrel, which is prevalent in up to 30% in some patient populations.^19^

While these features are desirable in the setting of staged CAS-CTS, several risks and limitations of the drug must be considered. Though not demonstrated with eptifibatide specifically, small studies have reported higher rates of intracerebral hemorrhage with other GIIBIIIA parenteral agents.^20^ Another rare, but serious complication of eptifibatide is severe thrombocytopenia which could have catastrophic consequences in patients undergoing CTS.^21^ The current study found a minor bleeding complication rate of 9.1%, comparable to another recent series of 16 patients treated with eptifibatide for ‘urgent’ CAS (groin hematoma in 6.2%).^22^ The cost-effectiveness of eptifibatide bridging is difficult to estimate from the current data. Previous studies have demonstrated relative cost-savings for percutaneous coronary intervention with eptifibatide compared to abciximab.^23^ Post-acute benefits of eptifibatide in terms of reduced complications and re-intervention have also been shown with respect to quality-adjusted-life-years^24^ and overall costs to third-party payers, all prior to the introduction of a generic formulation of the drug in 2016.^25^ Cangrelor (*Kengreal*), a newer, intravenous P2Y12 inhibitor with a half-life of 3–5 min is another candidate synergistic dual antiplatelet bridging agent which merits discussion.^26^ In randomized studies, *cangrelor* outperformed clopidogrel in reducing acute thrombogenic complications of coronary intervention and mortality without excess bleeding.^27^ *Cangrelor* has also been validated as a well-tolerated, effective pre-CABG bridging antiplatelet agent in randomized evaluation.^28^ Given its pharmacologic versatility, *cangrelor* is an exciting potential tool for transient platelet inhibition necessary for CAS-CTS and the authors look forward to future study of this agent in this population.

It is important to acknowledge that staged CAS-CTS is one of several revascularization strategies and the described bridging antiplatelet protocol is likely not a “one size fits all” solution. To date, there are no randomized studies comparing combination ICA revascularization strategies for patients undergoing combined carotid revascularization and CTS with the exception of the discontinued CABACS trial which investigated CEA only.^29^ In select high-risk patients, CAS may be favorable to CEA when procedural complication rates are very low (<3%).^30^ However, those older than 80 years or with challenging anatomy (concentric calcification, multiple sharp bends) have higher rates of procedural complications with CAS relative to CEA.^30^ Considering that the post-operative stroke risk following CTS is likely higher than natural history (over a similar 30-day period), a slightly higher complication rate may be tolerable in staged CAS-CTS. A meta-analysis of over 27,000 patients showed lower post-operative stroke for CAS compared with CEA staged procedures (2.4 vs. 3.9%, OR 1.62 CI 1.1–2.5, *p* =0.02) but no differences in mortality.^31^ However, a more recent inclusive meta-analysis contradicted this report, showing similar post-operative stroke risks (3%) but lower post-operative death rates in CAS relative to CEA.^5^ Other meta-analyses and retrospective series have reported small, but significant improved outcomes in CAS relative to CEA when outcomes beyond the 30 -day perioperative period are studied.^7,32,33^ Thus while staged CAS-CTS may be appropriate for some patients, CEA-revascularization strategies, or even medical therapy may be appropriate for many patients with co-morbid ICA and cardiac disease.^1,2,34^ For such cases in which CAS-CTS may be favorable, the eptifibatide bridging protocol affords a safe pharmacologic strategy which offers greater flexibility than previous strategies when combined treatment is desired during a single hospitalization.

The chief limitation of this study is the small, relatively homogenous patient sample. The generalizability of the findings should therefore be interpreted cautiously. Similarly, the study is underpowered to detect statistically meaningful differences in outcomes relative to conventional antiplatelet strategies. Nevertheless, the low rates of complication during the interlude and post-operative period provide valuable preliminary safety data to support continuation of the bridging protocol and merit adoption of this practice at other sites which treat patients with combined cardiac/carotid disease.

In summary, this single-center retrospective series of an eptifibatide bridging therapy strategy for staged CAS and CTS shows that this approach is safe and feasible with an acceptable minor bleeding risk. For select patients with clinical and anatomic characteristics which favor this revascularization strategy, the bridging antiplatelet protocol confers greater flexibility in surgical timing with low complication rates.
